# Reactivation of the Tumour Suppressor *RASSF1A* in Breast Cancer by Simultaneous Targeting of DNA and E2F1 Methylation

**DOI:** 10.1371/journal.pone.0052231

**Published:** 2012-12-14

**Authors:** María F. Montenegro, Magali Sáez-Ayala, Antonio Piñero-Madrona, Juan Cabezas-Herrera, José Neptuno Rodríguez-López

**Affiliations:** 1 Department of Biochemistry and Molecular Biology A, School of Biology, Regional Campus of International Excellence “Campus Mare Nostrum”, University of Murcia, Espinardo, Murcia, Spain; 2 Department of Surgery, University Hospital Virgen de la Arrixaca, Instituto Murciano de Investigación Biomédica, Murcia, Spain; 3 Translational Cancer Research Group, University Hospital Virgen de la Arrixaca, Instituto Murciano de Investigación Biomédica, Murcia, Spain; H.Lee Moffitt Cancer Center & Research Institute, United States of America

## Abstract

**Background:**

Tumour suppressor genes are often transcriptionally silenced by promoter hypermethylation, and recent research has implicated alterations in chromatin structure as the mechanistic basis for this repression. In addition to DNA methylation, other epigenetic post-translational modifications that modulate the stability and binding of specific transcription factors to gene promoters have emerged as important mechanisms for controlling gene expression. The aim of this study was to analyse the implications of these mechanisms and their molecular connections in the reactivation of *RASSF1A* in breast cancer.

**Methods:**

Compounds that modulate the intracellular concentration of adenosine, such as dipyridamole (DIPY), greatly increase the antiproliferative effects of 3-*O*-(3,4,5-trimethoxybenzoyl)-(−)-catechin (TMCG), a synthetic antifolate derived from the structure of tea catechins. Quantitative real-time PCR arrays and MALDI-TOF mass spectrometry indicated that this combination (TMCG/DIPY) induced apoptosis in breast cancer cells by modulating the methylation levels of DNA and proteins (such as E2F1), respectively. Chromatin immunoprecipitation (ChIP) assays were employed to confirm that this combination induced chromatin remodelling of the *RASSF1A* promoter and increased the occupancy of E2F1 at the promoter of this tumour suppressor gene.

**Results:**

The TMCG/DIPY combination acted as an epigenetic treatment that reactivated *RASSF1A* expression and induced apoptosis in breast cancer cells. In addition to modulating DNA methylation and chromatin remodelling, this combination also induced demethylation of the E2F1 transcription factor. The ChIP assay showed enhancement of E2F1 occupancy at the unmethylated *RASSF1A* promoter after TMCG/DIPY treatment. Interestingly, inhibition of E2F1 demethylation using an irreversible inhibitor of lysine-specific demethylase 1 reduced both TMCG/DIPY-mediated *RASSF1A* expression and apoptosis in MDA-MB-231 cells, suggesting that DNA and protein demethylation may act together to control these molecular and cellular processes.

**Conclusions/Significance:**

This study demonstrates that simultaneous targeting of DNA and E2F1 methylation is an effective epigenetic treatment that reactivates *RASSF1A* expression and induces apoptosis in breast cancer cells.

## Introduction

Breast cancer, like all cancers, is thought to result in part from the accumulation of genetic alterations that lead to oncogene overexpression and tumour suppressor loss. Substantial experimental evidence has documented the association between CpG island methylation and gene transcriptional inactivity, but researchers have only recently begun to discover the underlying mechanisms of transcriptional silencing by methylation. One possible mechanism of transcriptional repression is direct interference with the binding of sequence-specific transcription factors (such as AP-2, E2F and NFκB) to DNA, through methylation [1]. Recently, chromatin structure has emerged as an important and more generalised mechanism for silencing a variety of methylated tissue-specific and imprinted genes by histone deacetylase (HDAC) family members [2], [3]. The deacetylation of histone H3 and H4 lysine groups allows ionic interactions between positively charged lysines and negatively charged DNA, resulting in a more compact nucleosome structure that limits gene activity. The discovery of the family of methyl-CpG-binding proteins (such as MeCP2) provides a mechanistic link between DNA methylation and histone deacetylation as mediators of gene transcription. Common functional features of these proteins include their binding to methyl-CpGs in DNA and frequent association with members of the HDAC family, which currently includes eight distinct members [4]. These processes may collaborate to regulate gene expression, and studies have shown that multiple hypermethylated genes can be robustly reactivated by a combination of DNA-methyltransferase-1 (DNMT1) and HDAC inhibition, suggesting that DNMT1 and HDAC are both essential in the silencing of gene expression in cancer cells [3], [5].

In addition to CpG island methylation, the methylation status of transcription factors (such as E2F1) has also been overlooked as an additional mechanism that controls gene expression [6]–[9]. Therefore, the importance of these epigenetic mechanisms in controlling the expression of specific genes in cancer suggests that targeting of the methionine cycle in cancer cells may represent an attractive strategy for developing therapies that reactivate tumour suppressors in these cells [3], [10]. To design such therapies, it is important to consider the well-established connection between the methionine cycle and two crucial cell metabolites, folic acid and adenosine (Fig. S1). Folic acid acts as the fuel for the methionine cycle; after transformation by folate cycle enzymes [such as dihydrofolate reductase (DHFR), thymine synthase (TS) and 5,10-methylene-tetrahydrofolate reductase (MTHFR)], folic acid forms N^5^-methyl-tetrahydrofolate (N^5^-CH_3_-THF), the cofactor for methionine synthase (MS), which is the enzyme responsible for methionine synthesis. In contrast, adenosine is a product of the methionine cycle and is produced at high concentrations in tumour cells. The efficient intracellular elimination of this product by adenosine-transforming enzymes, such as adenosine deaminase (ADA), or its transport out of the cells by specific adenosine transporters, such as the equilibrate nucleoside transporters (ENTs), is of vital importance for cancer cell survival. Recently, we have observed that a combined therapy designed to uncouple adenosine metabolism using dipyridamole (DIPY) (an effective inhibitor of both ENTs and ADA) in the presence of a new synthetic antifolate [3-*O*-(3,4,5-trimethoxybenzoyl)-(−)-epicatechin; TMECG] simultaneously and efficiently blocked both the folic and methionine cycles in melanoma cells and resulted in massive cell death [10]. TMECG, a tyrosinase-processed antifolate prodrug, was shown to be active exclusively in melanoma [11]. However, its catechin epimer derivative, 3-*O*-(3,4,5-trimethoxybenzoyl)-(−)-catechin (TMCG), showed substantial antiproliferative activity in other epithelial cancer cell lines, including breast cancer cells [12]. Therefore, the aim of this study was to evaluate whether the combination of TMCG and DIPY represents a valuable epigenetic therapy against breast cancer. In addition, to study the effect of this combination on the methylation status of breast cancer cells, we also examined the reactivation of expression of RAS-association domain family 1, isoform A (*RASSF1A*), a tumour suppressor gene pathway that can regulate proliferation, induce apoptosis, and bind to and stabilise microtubules [13]. Inactivation of *RASSF1A* is frequently observed in multiple solid tumours and epithelial cancers, including breast cancer [5], [14], [15]. Because the gene remains intact but dormant in most tumours, reactivation by promoter demethylation would present a novel approach to therapy [13].

## Results

### Synergistic Effect of Simultaneous Treatment with TMCG and DIPY on Breast Cancer Cells

**Figure 1:**
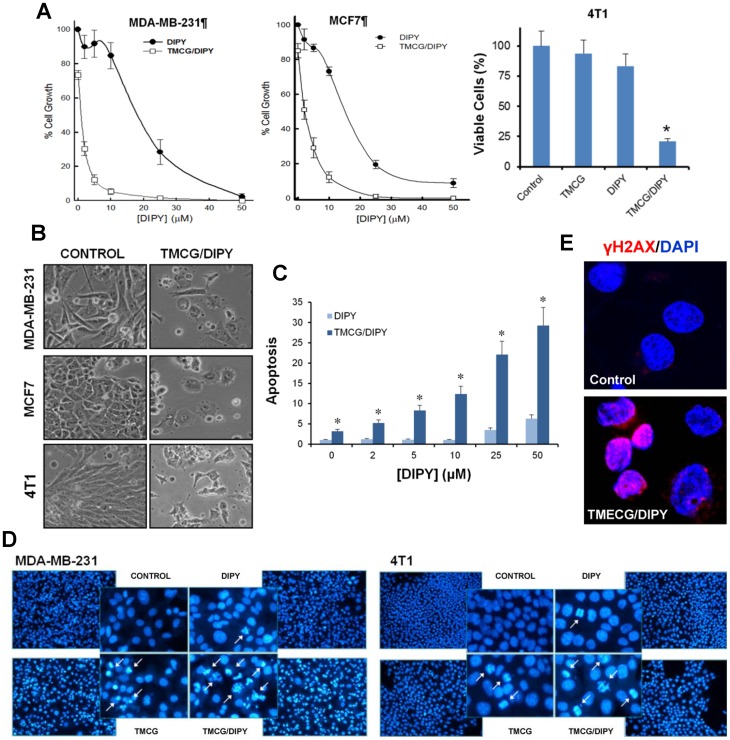
TMCG and DIPY act synergistically to induce apoptosis in breast cancer cells. (A) The left and central panels represent the effects of various DIPY concentrations on MDA-MB-231 and MCF7 cell growth, respectively, after 3 days of treatment in the absence (DIPY series; •) or the presence of 10 µM TMCG (TMCG/DIPY series; □). The right panel represents 4T1 cells that were treated for 3 days with vehicle only (DMSO; control), DIPY (5 µM), TMCG (10 µM) or a combination of the same concentration of DIPY plus TMCG. In all cases, the data are expressed relative to 100% growth or viability of the untreated control cells. *P<0.05 compared with single TMCG and DIPY treatments. (B) Morphology of untreated breast cancer cells (control) compared with those subjected to 3 days of treatment with 10 μM TMCG and 5 μM DIPY (TMCG/DIPY). (C) The histograms represent the effects of DIPY and TMCG/DIPY treatment on apoptosis in MCF7 cells (*P<0.05 relative to DIPY-treated cells). The dose-dependent effects after 3 days of treatment with DIPY were analysed using increasing concentrations of DIPY in the absence (DIPY series) or the presence of 10 μM TMCG (TMCG/DIPY series). (D) Apoptosis induction in MDA-MB-231 and 4T1 breast cancer cells after 3 days of treatment with vehicle (control), DIPY (5 μM), TMCG (10 μM) or a combination of the same concentration of DIPY plus TMCG was visualised by fluorescence microscopy of DNA stained with Hoechst 33342. The arrows indicate apoptotic cells. (E) MDA-MB-231 cells treated for 3 days with vehicle (control) or a combination of 10 µM TMCG and 5 µM DIPY (TMCG/DIPY) were examined for γH2AX nuclear foci (red). The nuclei were counterstained with DAPI (blue), and merged images are shown.

As shown in Fig. 1A, treatment of MDA-MB-231 and MCF7 cells with DIPY inhibited cell growth, with calculated IC_50_ values (at 3 days) of 20 µM and 16 µM, respectively. Although TMCG also inhibited the growth of these cancer cells, its activity was more evident over longer treatment periods [12]; the growth of both MDA-MB-231 and MCF7 cells was less than 25% and 15%, respectively, of the growth of untreated controls after 3 days of treatment with a fixed concentration of 10 µM TMCG. However, in the presence of this concentration of TMCG, the IC_50_ value for DIPY dropped to 1.3 µM and 1.8 µM in MDA-MB-231 and MCF7 cells, respectively. The synergistic effect between TMCG and DIPY on the viability of the 4T1 mouse breast cancer cell line was also evident (Fig. 1A). Using bright field microscopy, we compared the antiproliferative action of 5 μM DIPY or 10 µM TMCG alone with that induced by a combination of the drugs (at the same concentration) after 3 days of treatment; important and significant differences were observed. Although treatment with DIPY or TMCG alone considerably reduced the number of cells, the cells appeared to be healthy, similar to the untreated control cells (Fig. S2). In contrast, breast cancer cells treated with the combination showed clear signs of cellular damage. The morphological changes included cell shrinkage, loss of cell–cell contact and fragmentation of the plasma and nuclear membranes (Fig. 1B).

### TMCG/DIPY Combination Induces Apoptosis in Breast Cancer Cells

These preliminary results indicated that sub-apoptotic concentrations of both TMCG and DIPY acted synergistically to induce apoptosis in breast cancer cells. To investigate whether the morphological changes induced by the TMCG/DIPY combination were due to apoptosis, MCF7 cells were treated for 3 days with fixed concentrations of the compounds, and the degree of apoptosis induction was evaluated using a DNA fragmentation assay (Fig. 1C). The results indicated that the reduced viability of breast cancer cells in the presence of TMCG/DIPY was indeed due to apoptosis induction (Fig. 1C). Apoptosis in MDA-MB-231 and 4T1 breast cancer cells was also confirmed by nuclear fragmentation, as visualised by fluorescence microscopy after DNA staining with Hoechst 33342 (Fig. 1D). Confocal microscopy also indicated that the TMCG/DIPY combination induced the formation of DNA double strand breaks characterised by phosphorylation of histone H2AX at Ser^139^ (γH2AX) [16]. Immunofluorescence revealed that combined TMCG/DIPY treatment of MDA-MB-231 cells led to the accumulation of γH2AX foci by 72 h (Fig. 1E), while single treatments with TMCG or DIPY alone did not induce massive DNA damage in this breast cancer cell line (data not shown). Although a larger number of breast cancer cell lines should be tested, this initial study suggests that the apoptotic effect of this combination might be independent of the mutational status of the *p53* gene and the levels of expression of the estrogen receptor-α (ERα), two genes/proteins that determine the sensitivity or resistance of breast cancer cells to apoptosis [17], [18]. In this respect, antiestrogen resistance often develops with prolonged exposure to hormone therapies, and is a major problem in the treatment of breast cancer. The absence of ERα is the cause of most common form of *de novo* resistance to tamoxifen. Therefore, and although the combination TMCG/DIPY was found to be effective on MDA-MB-231, an ERα negative breast cancer cell line, we were interested in investigate whether this combination was also active on tamoxifen-resistant cell lines developed by a prolonged exposition to this drug (acquired resistance). For this, we developed a tamoxifen-resistant cell line (MCF7TamR) by maintaining ER-positive breast cancer cells, MCF7, in 10 nM tamoxifen for more than six months to select for resistant phenotypes [19]. As observed in Fig. S3, MCF7TamR cells, when compared with the parenteral cell line, were highly resistant to 4-hydroxytamoxifen; however, the combination TMCG/DIPY induced a high degree of apoptosis in both cell lines. The therapeutic relevance of these findings will be discussed later.

### TMCG/DIPY Combination Modulates Gene Promoter Methylation in Breast Cancer Cells

**Figure 2:**
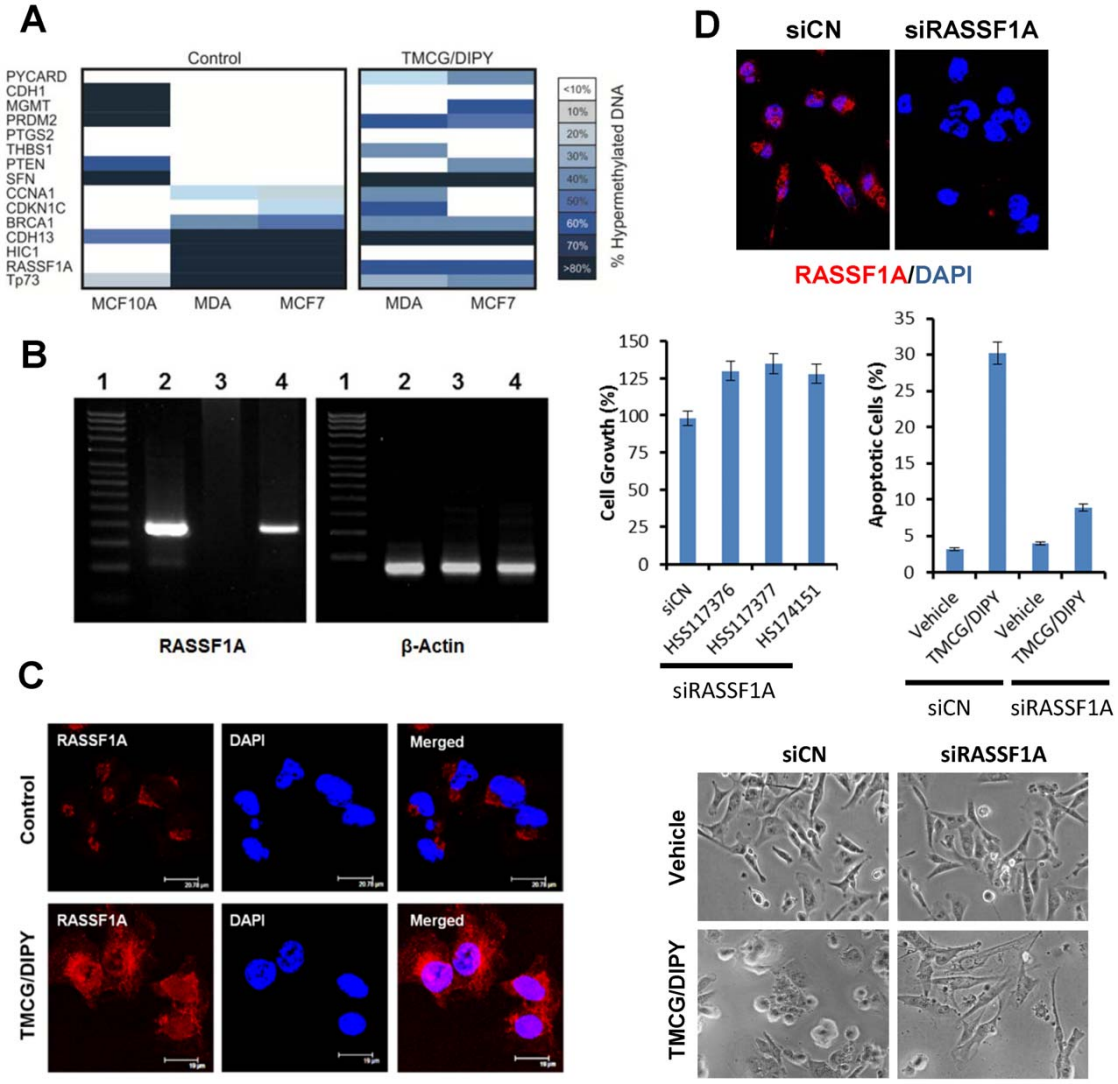
The TMCG/DIPY combination modulates gene promoter methylation in breast cancer cells and induces the expression of RASSF1A in MDA-MB-231 cells. (A) The methylation status of 15 gene promoters that have been reported to be altered in a variety of breast cancers as investigated by qRT-PCR array. Cells were treated for 3 days with vehicle (control) or with a combination of 10 μM TMCG and 5 μM DIPY (TMCG/DIPY). The MDA abbreviation indicates MDA-MB-231 cells. (B) Semiquantitative reverse transcription-PCR was used to detect RASSF1A (249 bp) and β-actin (142 bp) mRNA expression. Lane 1, DNA markers; Lane 2, expression in MCF10; Lane 3, expression in MDA-MB-231 cells treated for 3 days with vehicle; Lane 4, expression in MDA-MB-231 cells treated for 3 days with a combination of 10 μM TMCG and 5 μM DIPY. The data shown here are from a representative experiment repeated five times with similar results. (C) Immunohistochemical analysis of the expression of the RASSF1A protein in untreated MDA-MB-231 cells (control) and in cells treated for 3 days with a combination of 10 μM TMCG and 5 μM DIPY (TMCG/DIPY). The cells were stained with an anti-RASSF1A antibody (red) and DAPI (blue). Merged images are shown. (D) RASSF1A shRNA inhibits TMCG/DIPY-induced apoptosis in MDA-MB-231 cells. The effective silencing of RASSF1A was tested by confocal microscopy. shRNAs (HSS117376, HSS117377, and HSS174151) transfection significantly (*P*<0.05) increased MDA-MB-231 cell growth (the data, monitored 4 days after transfection, are expressed relative to 100% growth of the untransfected/untreated control cells). For TMCG/DIPY treatment, siCN- and siRASSF1A-transfected cells were treated for 3 days with a combination of 10 μM TMCG and 5 μM DIPY. Apoptosis was determined by fluorescence microscopy after DNA staining with Hoechst 33342. Differences in apoptosis between siCN- and siRASSF1A-TMCG/DIPY treated cells were statistically significant (*P*<0.05). Morphological changes before and after TMCG/DIPY treatments were visualised by bright field microscopy.

In addition to suppressing adenosine transport by inhibiting ENTs, DIPY also inhibits the enzyme ADA, which normally breaks down adenosine into inosine. In accordance with this activity, treatments of MDA-MB-231 with DIPY elevated intracellular adenosine in these cells (Fig. S4). Therefore, we hypothesised that DIPY-induced increase of adenosine, in the presence of TMCG-accumulated homocysteine, would produce an effective blockade of the methionine cycle [10]. To test this hypothesis, the methylation status of 24 gene promoters that have been reported to be altered in a variety of breast cancers was investigated using qRT-PCR arrays (Table S1). Genomic DNA was isolated from the non-tumorigenic breast cell line MCF10 and from two human breast cancer cell lines, MCF7 and MDA-MB-231. As expected, significant differences were observed in the methylation pattern between normal and cancer cells, and genes encoding three tumour suppressor proteins (HIC1, RASSF1A, and p73) displayed an increased percentage of promoter hypermethylation in cancer cells compared with non-tumour cells (Fig. 2A and Table S1). The effect of TMCG/DIPY treatment on the demethylation of the promoters of these genes was also evident (Fig. 2A and Table S1). For example, the *RASSF1A* gene promoter, which was highly methylated in MCF7 and MDA-MB-231 (90.37% and 87.98%, respectively), showed a significant reduction in methylation (*P*<0.05) to 60.01% and 62.91% in MCF7 and MDA-MB-231, respectively, after 3 days of TMCG/DIPY treatment. Collectively, these differences in DNA methylation indicated that the TMCG/DIPY combination efficiently decreased the methylation of the *HIC1*, *RASSF1A*, and *p73* promoters; this decreased methylation could lead to the direct binding of sequence-specific transcription factors and reactivation of gene expression.

### TMCG/DIPY Treatment Induces Chromatin Remodelling in MDA-MB-231 Breast Cancer Cells and Reactivates *RASSF1A* Expression

Although the biological role of each member of the RASSF group remains unclear, evidence exists suggesting that RASSF members serve as tumour suppressor genes by modulating some of the growth inhibitory responses mediated by Ras [20]. RASSF1A has been shown to play an important role in cell-cycle regulation, apoptosis, and microtubule stability [13], [21]. Indeed, ectopic expression of RASSF1A reduces the growth rate of human cancer cells, supporting a role for *RASSF1A* as a tumour suppressor gene [22]. Furthermore, the *RASSF1A* knockout mouse model shows an increased tendency to develop tumours [23]. The mechanisms by which RASSF1A acts as a tumour suppressor and the pathways that are involved are not yet fully understood. Because a functional relationship between DNA hypermethylation and the silencing of RASSF1A transcripts in breast cancer cells has been described [14], we next investigated whether demethylation of the *RASSF1A* promoter after TMCG/DIPY treatment was accompanied by an increase in expression of the RASSF1A transcript. To establish a functional relationship between DNA hypermethylation and RASSF1A transcript silencing, conventional PCR was performed in MDA-MB-231 breast tumour cells. In agreement with previous data [14], these transcripts were not detected in MDA-MB-231 cells, which showed high levels of *RASSF1A* promoter methylation (87.98%). In contrast, TMCG/DIPY-treated MDA-MB-231 cells showed a substantial increase in RASFF1A mRNA transcripts (Fig. 2B). The RASSF1A PCR product was sequenced, and the published RASSF1A sequence was confirmed (data not shown). Although RASSF1A mRNA was not detected in MDA-MB-231, confocal microscopy demonstrated the presence of the RASSF1A protein in these breast cancer cells (Fig. 2C). Because RASSF1 has microtubule-stabilising properties [13], its perinuclear localisation in untreated control cells suggests that this protein is associated with the microtubule network. However, upon treatment of MDA-MB-231 cells with TMCG/DIPY, the RASSF1A protein was observed in the nuclear and cytosolic compartments; thus, TMCG/DIPY treatment resulted not only in an increase in the RASSF1A protein but also in an altered cellular localisation (Fig. 2C). Next, to understand the role of RASSF1A in TMCG/DIPY-induced apoptosis, we analysed the response of MDA-MB-231 cells to this drug combination following shRNA-mediated silencing of RASSF1A (Fig. 2D). MDA-MB-231 cells were transfected with either shRNA-RASSF1A (siRASSF1A) or a control shRNA (siCN) and maintained in culture medium in the presence or absence of TMCG/DIPY. The results presented in Fig. 2D show that efficient downregulation of RASSF1A increased cell growth in the absence of drugs (comparing siRASSF1A to siCN), which agrees with its tumour suppressor function in breast cancer cells [13]. More importantly, depletion of RASSF1A significantly decreased the sensitivity of MDA-MB-231 cells to TMCG/DIPY-induced apoptosis. Although this drug combination induced important morphological changes in MDA-MB-231 silenced cells (cells were longer and narrower than normal or silenced untreated cells), apoptosis quantification assays indicated that RASSF1A was an important component of the apoptotic response of MDA-MB-231 to TMCG/DIPY-induced death.

**Figure 3:**
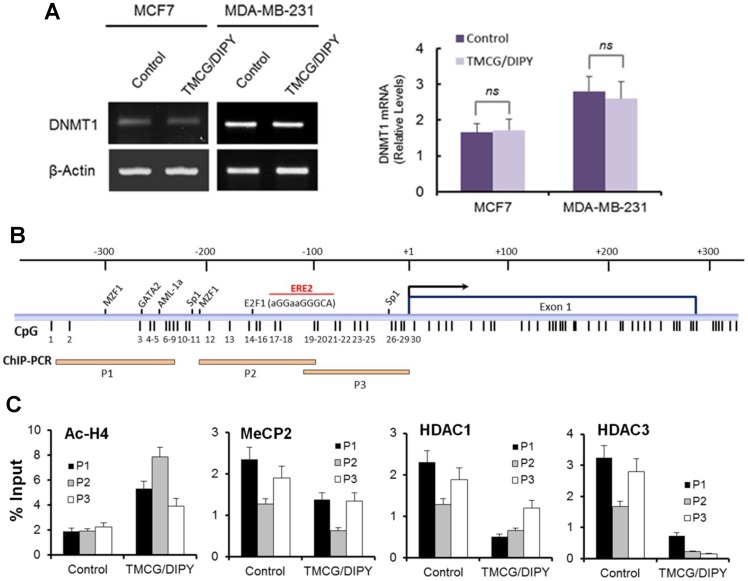
Inhibition of DNA methylation by TMCG/DIPY treatment induces chromatin remodelling in MDA-MB-231 breast cancer cells. (A) Semiquantitative determination of DNMT1 mRNA in breast cancer cells. The estimated relative levels of mRNA relative to that of β-actin were calculated and then compared to the expression levels in untreated controls. The histograms represent the number of copies of mRNA for every 1×10^3^ copies of β-actin. MCF7 and MDA-MB-231 cells were treated with vehicle (control) or a combination of 10 μM TMCG and 5 μM DIPY (TMCG/DIPY) for 3 days. There were no significant differences (*ns*) in DNMT1 mRNA expression between TMCG/DIPY-treated cells and untreated controls. (B) Genomic map of the *RASSF1A* CpG island. The positions of CpG sites in the genomic sequence are indicated by the thin vertical lines. Bent arrow: the position of the transcription start site. Potential transcription factor binding sites (MZF1, GATA2, AML-1a, and Sp1) resulting from the TFSEARCH query [14] are marked in the promoter region. The functional E2F binding site (ERE2, identified at site −150/-143 bp within the *RASSF1A* CpG island) [28] is also indicated. The bottom row of horizontal lines indicates the locations of *RASSF1A* promoter fragments used for the ChIP assay (P1, P2, and P3). (C) ChIP assay of the *RASSF1A* CpG island. Chromatin DNA was immunoprecipitated with antibodies specific for acetyl-H4, MeCP2, HDAC1, and HDAC3. DNA fragments corresponding to *RASSF1A* promoter regions P1, P2, and P3 were amplified by PCR. The histograms represent PCR analyses of ChIP in MDA-MB-231 in the three regions before and after 3 days of treatment with TMCG/DIPY (10 μM/5 μM). PCR was performed on immunoprecipitated DNA and total input DNA. The corresponding signal intensity enrichment of each ChIP-PCR analysis for the three regions is plotted. The enrichment levels are derived from fold changes between the normalised *RASSF1A* intensity in each immunoprecipitated DNA and the normalised *RASSF1A* intensity in the input DNA. In all cases, the changes after TMCG/DIPY treatment were statistically significant (*P*<0.05) compared with protein occupancies in untreated controls.

To evaluate whether TMCG/DIPY treatment modified the methylation status of DNA in breast cancer cells by interfering with expression of the methyltransferase DNMT1, the expression levels of this gene were analysed by qRT-PCR (Fig. 3A). TMCG/DIPY treatment did not affect DNMT1 expression in either MDA-MB-231 or MCF7 cells, suggesting that the demethylating activity of this combination was associated with DNMT1 enzyme inhibition, rather than DNMT1 expression. Because DNMT inhibitors (such as 5-aza-2′-deoxycytidine) have been shown to influence chromatin remodelling [5], we further analysed whether TMCG/DIPY treatment affects chromatin remodelling of the *RASSF1A* promoter using ChIP assays. Histone-associated DNA that was immunoprecipitated using an antibody against acetyl-H4 (Ac-H4) was amplified with three primer sets covering the *RASSF1A* promoter region (Fig. 3B) [14]. The results show that TMCG/DIPY treatment significantly affected the levels of histone acetylation on the *RASSF1A* promoter. An enhancement of histone acetylation was observed in TMCG/DIPY-treated MDA-MB-231 cells, in which the *RASSF1A* promoter was partially methylated and transcriptionally active; however, lower levels of acetylation of the same sites were observed in the hypermethylated, transcriptionally silenced promoter of untreated MDA-MB-231 cells (Fig. 3B). In agreement with these data, TMCG/DIPY treatment also reduced the occupancy of HDACs (1 and 3) and the methyl-CpG-binding protein MeCP2 at the *RASSF1A* promoter in MDA-MB-231 cells (Fig. 3B).

### TMCG/DIPY Treatment Demethylates E2F1 and Increases Its Occupancy at the *RASSF1A* Promoter

**Figure 4:**
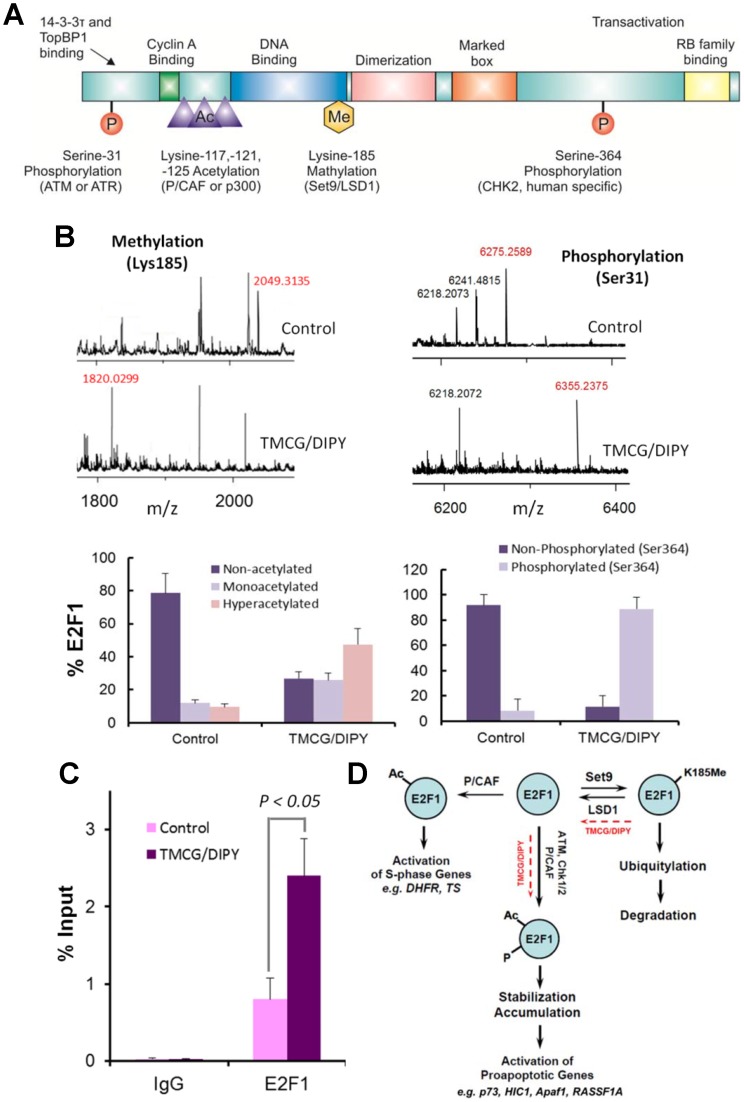
TMCG/DIPY modulates the posttranslational state of E2F1 and promotes its binding to the *RASSF1A* promoter. (A) Schematic representation of the E2F1 protein. Residues susceptible to methylation (K185), acetylation (K117, K120, and K125), and phosphorylation (S31 and S364) are shown. (B) MALDI-TOF mass spectra of tryptic digests of immunoprecipitated E2F1. Peptides were analysed in untreated MDA-MB-231 cells (control) or in those treated for 3 days with TMCG/DIPY (10 μM/5 μM). (C) *In vivo* binding of E2F1 to the RASSF1A promoter. ChIP assays using untreated MDA-MB-231 cells (control) or those treated for 3 days with TMCG/DIPY (10 μM/5 μM). The enrichment of the E2F1 protein on the *RASSF1A* promoter is shown. Immunoprecipitation (IP) using the anti-E2F1 or an IgG was performed in triplicate. The results are presented as a percentage of the input DNA. (D) The proposed mechanism for the regulation of E2F1. E2F1 is regulated by several posttranslational modifications, including methylation (Me), acetylation (Ac) and phosphorylation (P) [6]. The effect of TMCG/DIPY (red dashed line) on the E2F1 status is shown. E2F1 is reversibly methylated by the enzymatic actions of LSD1 and Set9.

The E2F family of transcription factors plays a key role in the regulation of cell growth, apoptosis, and oncogenic transformation by mediating the timely expression of genes involved in these processes [24]. E2F binding sites contain one or two CpG motifs and are therefore candidates for regulation by DNA methylation. While DNA methylation is one mechanism that regulates E2F activity, the methylation status of E2F1 protein, a member of the E2F family, has also been found to control both its stability and transcriptional activity. Recently, negative crosstalk between methylation and other post-translational modifications of E2F1, such as acetylation and phosphorylation, has been described (Fig. 4A) [6], [7]. Thus, methylated E2F1 is prone to ubiquitination and degradation, whereas the demethylation of E2F1 favours its P/CAF-dependent acetylation at lysine residues 117, 120, and 125 [25]. Whether acetylated E2F1 binds to the promoter of genes required for S phase (to allow cell growth) or to the promoters of proapoptotic genes (to induce cell death) may depend on its subsequent phosphorylation by specific kinases. Thus, in response to severe DNA damage, the hyperacetylated E2F1 protein is stabilised through direct phosphorylation by Chk2 at Ser^364^ or ATM kinase at Ser^31^ [26], [27]. Here, we observed that the TMCG/DIPY combination induced substantial changes in the posttranslational status of E2F1 in MDA-MB-231 cells. Mass peptide analysis of immunoprecipitated E2F1 after trypsin digestion indicated that TMCG/DIPY promoted the demethylation of E2F1 at Lys^185^ (Fig. 4B and Table 1). The specific modification of lysine side-chain amino groups, such as methylation or acetylation, results in completely different MS patterns after trypsin digestion of proteins. Trypsin digestion of E2F1 immunoprecipitated from untreated MDA-MB-231 control cells primarily yielded the peptide (K)SK_Me_NHIQWLGSHTTVGVGGR(L) (measured m/z  = 2049.3135; Table 1), which corresponded to the methylated Lys^185^-containing peptide. In contrast, treatment of MDA-MB-231 with a single dose of the TMCG/DIPY combination for 3 days greatly induced the demethylation of E2F1, which was confirmed by the higher proportion of the unmethylated peptide (K)NHIQWLGSHTTVGVGGR(L) (measured m/z of 1820.0299; Table 1). In agreement with the apoptotic data (which suggested that the TMCG/DIPY combination induced severe DNA damage in breast cancer cells), TMCG/DIPY-promoted demethylation of E2F1 was also accompanied by E2F1 acetylation and phosphorylation (at both Ser^31^ and Ser^364^) as determined by MALDI-TOF mass spectrometry (Fig. 4B and Table 1).

**Table 1:** MALDI-TOF mass spectroscopy properties of immunoprecipitated E2F1 tryptic digests.

Modification	E2F1 Status	Peptide Sequence^a^	Measured^a ^(*m/z*)	Theoretical^a ^(*m/z*)	Control^b^	TMCG/DIPY^b^(Intensity)^c^	TMCG/DIPY/2PCPA^b^
Methylation (K185)	Non-methylated	(K)NHIQWLGSHTTVGVGGR(L)	1820.0299	1820.0331	15	212	16
	Methylated	(K)SK**_Me_**NHIQWLGSHTTVGVGGR(L)	2049.3135	2049.3140	155	18	213
Acetylation (K117,120,125)	Non-acetylated Mono-acetylated	(K)SPGEK(S)	517.5611	517.5625	206	167	296
		(K)GVK_Ac_SPGEK(S)	801.9227	801.9233	31	161	21
	Hyperacetylated	(R)HPGK**_Ac_**GVK**_Ac_**SPGEK**_Ac_**SR(Y)	1589.8399	1589.8394	25	296	23
Phosphorylation (S31)	Non-phosphorylated	(R)LLDSSQIVIISAAQDASAPPAPTGPAAPAAGPC(Carbamidomethyl)DPDLLLFATPQAPRPTPSAPRPALGRPPVK(R)	6275.2592	6275.2600	116	15	209
	Phosphorylated	(R)LLDSS**_P_**QIVIISAAQDASAPPAPTGPAAPAAGPC(Carbamidomethyl)DPDLLLFATPQAPRPTPSAPRPALGRPPVK(R)	6355.2361	6355.2398	11	112	21
Phosphorylation (S364)	Non-phosphorylated	(R)MGSLRAPVDEDR(L)	1346.5131	1346.5139	507	31	448
	Phosphorylated	(R)MGS**_P_**LRAPVDEDR(L)	1426.4931	1426.4937	46	244	23

a The characteristics peptides involving posttranslational modifications of E2F1 (methylation  =  Me, acetylation  =  Ac, and phosphorylation  = P), as well as their measured and theoretical *m/z* are shown; ^b^Peptides were analyzed in untreated MDA-MB-231 cells (control), treated for 3-days with 10 μM TMCG plus 5 μM DIPY (TMCG/DIPY) or treated for 3-days with 10 μM TMCG plus 5 μM DIPY in the presence of 50 μM 2PCPA (TMCG/DIPY/2PCPA); ^c^Relative intensities of specific tryptic peptides were normalized with respect to an internal matrix control.

E2F1 acetylation has been observed to be requisite for binding to specific gene promoters [25]. Because E2F1 seems to be necessary for full expression of RASSF1A after treatment with TMCG/DIPY (Fig. S5), we next investigated whether demethylation/acetylation of this transcription factor increased its occupancy at the *RASSF1A* promoter after TMCG/DIPY treatment. Recently, Chang et al. [28] have found three putative E2F1-binding sites within the *RASSF1A* CpG island (at −150/−143, −5/+3 and +13/+23 bp; Fig. 3B), identifying this tumour suppressor gene as a transcriptional target of E2F1. This study also confirmed that hypermethylation at the CpGs located within the E2F1 binding sites blocks E2F1 binding and suppresses *RASSF1A* expression in the A549 human lung cancer cell line [28]. To address whether the transcription factor E2F1 binds to the identified putative E2F1 binding sites within the *RASSF1A* promoter *in vivo*, we performed a ChIP assay in TMCG/DIPY-treated MDA-MB-231 cells or untreated MDA-MB-231 cells using the P2 set of primers (Fig. 4C). Compared with untreated cells, TMCG/DIPY treatment significantly increased the occupancy of E2F1 at the *RASSF1A* promoter of MDA-MB-231 (from 0.8% in untreated cells to 2.4% in cells treated with TMCG/DIPY for 72 h relative to an input control).

### E2F1 Demethylation Contributes to TMCG/DIPY-induced Apoptosis and *RASSF1* Activation in MDA-MB-231 Breast Cancer Cells

**Figure 5:**
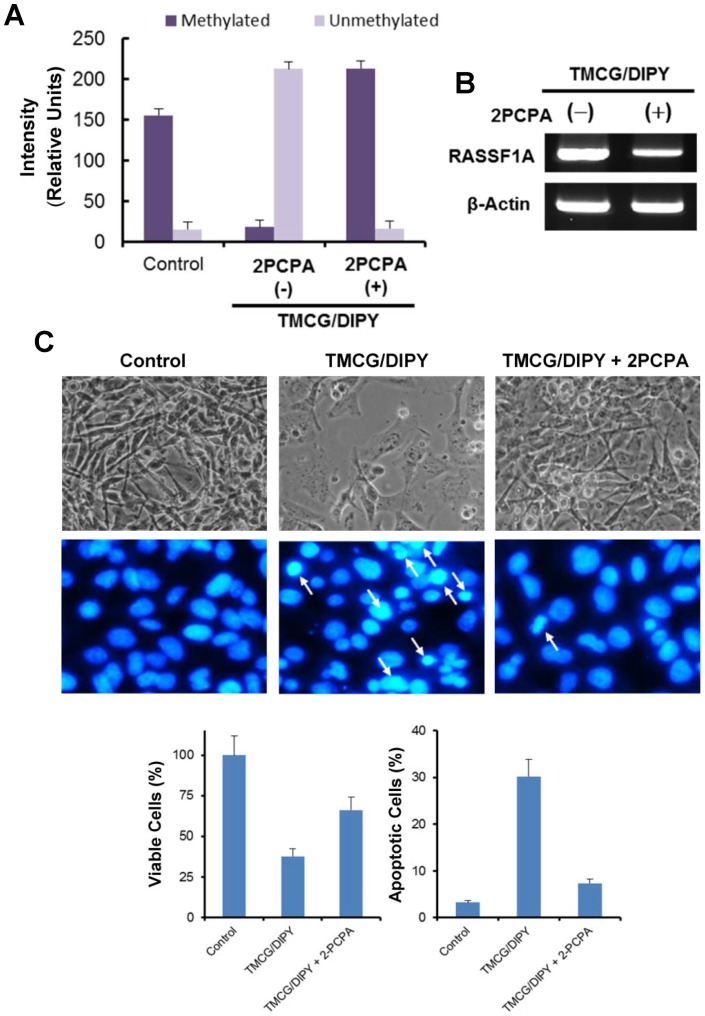
Effect of LSD1 inhibition on TMCG/DIPY activity in MDA-MB-231 breast cancer cells. (A) Relative intensity of methylated [(K)SK_Me_NHIQWLGSHTTVGVGGR(L); *m/z* 2049.3135] and unmethylated [(K)NHIQWLGSHTTVGVGGR(L); *m/z* 1820.0299] peptides in E2F1-trypsin digested samples. Peptides were analysed in MDA-MB-231 cells treated for 3 days with vehicle (control) or 10 µM TMCG and 5 µM DIPY in the absence (−) or the presence (+) of 50 µM 2PCPA (*P<0.05 compared with TMCG/DIPY treatments). The intensities were normalised with respect to an internal matrix control. (B) Semiquantitative reverse transcription-PCR was used to detect RASSF1A mRNA in MDA-MB-231 cells treated for 3 days with 10 µM TMCG and 5 µM DIPY in the absence (−) or the presence (+) of 50 µM 2PCPA. β-actin was used as a loading control. (C) 2PCPA inhibits TMCG/DIPY-induced apoptosis in MDA-MB-231 cells. The images show the effect of TMCG/DIPY or TMCG/DIPY/2PCPA treatment on the morphology (upper panels) and apoptosis (lower panels) of MDA-MB-231 cells as determined by bright field microscopy and fluorescence microscopy after DNA staining with Hoechst 33342, respectively. The histograms represent the percentage of viable and apoptotic cells calculated from the images shown above. *P<0.05 compared with untreated controls; **P<0.05 compared with TMCG/DIPY treatment. In all cases, the cells were treated with vehicle (control), 10 µM TMCG and 5 µM DIPY (TMCG/DIPY), or 10µM TMCG, 5µM DIPY, and 50µM 2PCPA (TMCG/DIPY/2PCPA).

A recent study has proposed that Set9 and LSD1 differentially influence DNA damage-induced cell death in tumour cells [6], [7]. Set9-mediated methylation of E2F1 destabilises the protein and negatively influences E2F1-mediated cell death, whereas LSD1-mediated demethylation of E2F1 is required for DNA damage-induced accumulation of this transcription factor and activation of apoptotic targets (Fig. 4D). Therefore, to analyse whether demethylation of E2F1 is a key element in TMCG/DIPY-induced signalling, we analysed the effect of 2PCPA, an irreversible LSD1 inhibitor, on the activity of TMCG/DIPY in these cells. First, we confirmed whether 2PCPA was able to inhibit the TMCG/DIPY-induced demethylation of E2F1 in MDA-MB-231 cells using MALDI-TOF mass spectrometry (Fig. 5A and Table 1). LSD1 inhibition in the presence of TMCG/DIPY resulted in an effective blockage of E2F1 demethylation (Fig. 5A and Table 1). As expected, low levels of unmethylated E2F1 were accompanied by a significant reduction in the levels of the acetylated and phosphorylated forms of this transcription factor compared with the levels observed in TMCG/DIPY-treated cells (Table 1). In contrast to the observed effects on E2F1 methylation, qRT-PCR arrays revealed that 2PCPA did not influence TMCG/DIPY-induced demethylation of the *RASSF1A* promoter (62.91% and 62.28% of DNA methylation when treated with TMCG/DIPY and TMCG/DIPY/2PCPA, respectively). These results provided the opportunity to study the contribution of E2F1 versus DNA demethylation to two TMCG/DIPY-mediated processes in breast cancer cells, *RASSF1A* reactivation and apoptosis induction.

PCR experiments clearly indicated that the triple TMCG/DIPY/2PCPA combination significantly reduced the expression of RASSF1A mRNA by 64%±8% compared with its expression in MDA-MB-231 cells treated with TMCG/DIPY only (Fig. 5B). These results highlighted the involvement of E2F1 in RASSF1A transcription. However, the observation that complete demethylation of the E2F1 protein by 2PCPA (Fig. 5A) only partially reduced *RASSF1A* promoter activity suggests that after demethylation of the *RASSF1A* promoter by TMCG/DIPY treatment, other activators (such as SP1) might also be involved in the transcriptional activation of the *RASSF1A* gene [28]. Finally, we analysed whether LSD1 inhibition interfered with TMCG/DIPY-induced apoptosis. As shown in Fig. 5C, 2PCPA strongly reversed the effect of TMCG/DIPY on MDA-MB-231 cell morphology and apoptosis (as determined by fluorescence microscopy after DNA staining with Hoechst 33342). The results agree with those obtained by shRNA-mediated silencing of E2F1 (Fig. S5) and indicate that TMCG/DIPY may induce E2F1-mediated apoptosis in breast cancer cells [29].

## Discussion

The limitations of conventional chemotherapy treatments, especially for patients with advanced cancer, have become apparent. New therapeutic strategies must be identified, and the metabolic abnormalities of cancer cells provide opportunities for alternative strategies [30]. Many human cancer cells lines and primary tumours have an absolute need for methionine, an essential amino acid. In contrast, normal cells are relatively resistant to exogenous methionine restriction. In this study, we show that breast cancer cells are highly dependent on methionine. The resistance of cancers to general chemotherapeutics and their evasion of cellular suicide and resistance to apoptosis are primarily related to the high activity of the methionine cycle in these cells, which permits the methylation of specific genes and activation of multiple survival pathways. Blocking the methionine cycle using the combination of a novel synthetic antifolate, TMCG, and a compound that uncouples adenosine metabolism, DIPY, seems to represent an effective therapy against breast cancer. Our results show that this combination has potential antitumour activity because these agents modulate multiple aspects of breast cancer cell metabolism and survival, including the folic acid and methionine cycles and the methylation status of cells. This broad spectrum of antitumour activities, in conjunction with low toxicity, underlies the translational potential of this combination for use as part of a therapeutic strategy against breast cancer.

In addition to the promising therapeutic properties of the TMCG/DIPY combination, we described in this study a coordinate mechanism by which simultaneous demethylation of DNA and E2F1 contributes to the reactivation of the tumour suppressor *RASSF1A* in breast cancer cells. As described in the introductory section, adenosine is a direct product of the methionine cycle and is produced at high concentrations when the cycle is highly active. Any resulting excess of adenosine may not present a problem for cancer cells. Adenosine is efficiently metabolised by specific enzymes (such as ADA and adenosine kinase) before use in purine nucleotide synthesis, which is particularly necessary for DNA synthesis in these highly proliferating cells. Furthermore, excess adenosine can be transported out of the cells by ENTs, which are bidirectional transporters that allow adenosine release and uptake by facilitating diffusion along its concentration gradient. However, in the presence of an antifolate compound, adenosine accumulation may represent a severe problem for the cell. In folate-deficient cells (including cells treated with antifolates), depletion of N^5^-methyl-THF blocks the methylation of homocysteine. The resulting accumulation of homocysteine drives S-adenosylhomocysteine hydrolase (AHYC) to catalyse the energetically favourable reverse reaction and synthesise S-adenosylhomocysteine (SAH), a potent product inhibitor of cellular methyltransferases (Fig. S1). This decrease in methylation has been overlooked as a mechanism for the antiproliferative effects of antifolates [31]. According to this mechanism of action, the treatment of breast cancer cells with TMCG/DIPY would result in a broad indirect SAH-mediated inhibition of cellular methylases. Although specific inhibition of DNMTs primarily results in unmethylated DNA, TMCG/DIPY can inhibit both DNA and protein methylation. Protein methyltransferases (PMTs), which methylate lysine or arginine residues on histones and other proteins, are emerging as an important group of enzymes that play key roles in normal physiology and human diseases [32]. Among these enzymes is Set9, a histone methyltransferase, which efficiently methylates p53 and E2F1 at Lys^372^ and Lys^185^, respectively, with opposite consequences on their respective activities [6], [33]. Thus, although Set9-mediated methylation of E2F1 destabilises the protein and impedes E2F1-mediated apoptosis, methylation of p53 by Set9 stabilises this transcription factor, which promotes apoptosis [6].

**Figure 6:**
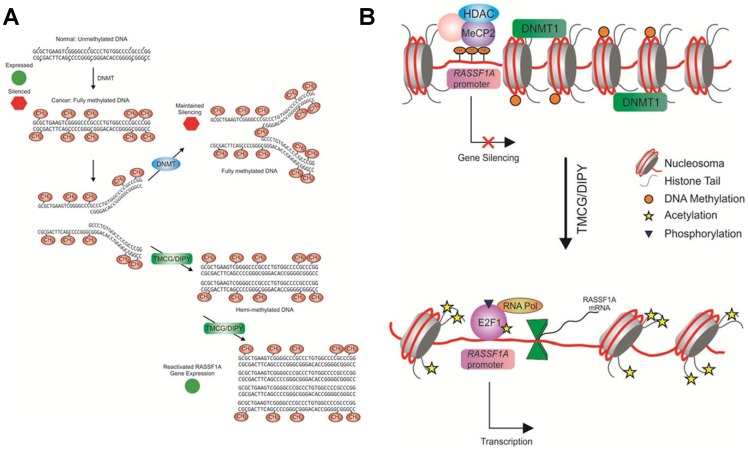
Proposed models for the reactivation of *RASSF1A* **by the TMCG/DIPY combination.** (A) Schematic representation of the effect of TMCG/DIPY on DNA methylation. Inhibition of DNMTs by the TMCG/DIPY-induced depletion of SAH favours DNA demethylation during replication. (B) Inhibition of DNMTs by the TMCG/DIPY combination may result in the remodelling of chromatin in the region of the *RASSF1A* promoter into a transcriptionally permissive state, which may permit the binding of transactivated E2F1 (and other transcription factors) and synthesis of the RASSF1A transcript.

Therefore, based on the results of this study, we can summarise the global mechanism for TMCG/DIPY action as follows. On the one hand, indirect inhibition of DNMTs by the TMCG/DIPY combination prevents DNA methylation following DNA replication (Fig. 6A), which may result in chromatin remodelling in the region of the *RASSF1* promoter into a transcriptionally permissive state [28] (Fig. 6B). We observed changes in both histone H4 acetylation and in the occupancy of histone modifying enzymes (HDACs) or methyl-CpG-binding proteins (such as MeCP2) at the *RASSF1A* promoter upon inhibition of DNA methyltransferase. These changes suggest that in breast cancer cells, DNA hypermethylation (or another activity mediated by DNMTs) may be essential for maintaining repressive histone modifications at gene promoters silenced by aberrant DNA hypermethylation. Furthermore, the observation that TMCG/DIPY can both reactivate the expression of the silenced *RASSF1A* gene and reverse key histone modifications surrounding the gene promoter strengthens the idea that these two events are interdependent. The E2F1 transcription factor is well known to be unable to bind to a recognition sequence that contains methylated CpG dinucleotides [24]; therefore, after TMCG/DIPY treatment, DNA demethylation and changes in the chromatin structure in downstream sequences of the *RASSF1A* gene are essential for the observed binding of E2F1 to potential transcriptionally important CpG sites of the *RASSF1A* promoter (Fig. 6B). On the other hand, in addition to DNA methylation, TMCG/DIPY-induced protein demethylation may also play a crucial role in the E2F1-dependent expression of the RASSF1A transcript. Indirect inhibition of Set9 by the TMCG/DIPY combination disturbs the equilibrium between methylated/demethylated E2F1, favouring the cellular presence of the unmethylated form of this transcription factor (Fig. 4D). The results presented in this study suggest a dynamic interplay between methylation and other posttranslational modifications of E2F1 in breast cancer cells, in which the *in vivo* levels of methylated and unmethylated E2F1 are determined by the opposing actions of Set9 and LSD1 [6], [7]. Methylation of E2F1 has been determined to preclude its acetylation and phosphorylation at distant amino acids, which are required for DNA damage-induced stabilisation of the protein and activation of proapoptotic target genes. Therefore, the results showing that inhibition of LSD1 in the presence of TMCG and DIPY reduced both RASSF1A mRNA expression and apoptosis in MDA-MB-231 cells indicate that unmethylated E2F1 is required not only for the binding of E2F1 to the *RASSF1A* promoter but also for TMCG/DIPY-induced apoptosis in these breast cancer cells.

After its stabilisation by acetylation and phosphorylation, E2F1 controls the expression of many proapoptotic genes, such as *p73*, *Apaf1, HIC1,* and *RASSF1A,* among others. Therefore, the observation that 2PCPA inhibited TMCG/DIPY-induced apoptosis in MDA-MB-231 cells indicates that this apoptotic process may be coordinated by a set of proapoptotic proteins. In fact, in addition to *RASSF1A*, the promoters of two other tumour suppressor genes and well-characterised E2F1 targets [26], [34], *HIC1* and *p73*, were also found to be highly demethylated after TMCG/DIPY exposure (Fig. 2A). The RASSF1A tumour suppressor protein has been observed to function in a coordinated manner with the product of another E2F1 target gene, p73, to elicit apoptosis through the proapoptotic mammalian STE20-like kinases MST2 pathway [35]. Indications that MST1/2 pathways might be involved in RASSF1A-mediated apoptosis have also recently been described by Guo et al. [36]. These authors have shown that the RASSF1A protein functions in MST kinase pathways to provide and preserve the phosphorylated/active state of MST1 and MST2 by preventing dephosphorylation of these kinases by the protein phosphatase PP2A. This discovery illustrates the great complexity in cell apoptotic pathways. Intriguingly, PP2A, a phosphatase that prevents MST1/2-induced apoptosis by competing with RASSF1A, is also controlled by protein methylation. PP2A is a trimeric serine/threonine phosphatase that contains a regulatory subunit B, which is recruited by a C-A dimer composed of the catalytic subunit C (PP2AC) and a structural subunit A [37]. Recruitment occurs when C is carboxyl-methylated on the terminal Leu^309^, which results in the assembly of the active PP2A trimer. Thus, changes in PP2A methylation can modulate the specificity and activity of PP2A in cells. Reversible methylation of PP2A is catalysed by two conserved and PP2A-specific enzymes, leucine carboxyl methyltransferase (LCMT1) and PP2A methylesterase [38], [39]. Because LCMT1 is a specific SAM-dependent methyltransferase, it is tempting to speculate that an increase in the cellular SAH after the treatment of breast cancer cells with TMCG/DIPY may also result in the inhibition of PP2A assembly. Thus, the absence of PP2A activity in TMCG/DIPY-treated cells would facilitate MST kinases-RASSF1A-mediated apoptosis [36]. Although additional studies are needed to completely understand the mechanisms by which the TMCG/DIPY combination induces apoptosis in breast cancer cells, another important observation derived from this study is that this combination induced apoptosis not only in MCF7, a cell line that expresses only wild-type p53, but also in MDA-MB-231 and 4T1 cells, which harbour mutant p53. Therefore, the results indicate that the drugs cooperate to induce p53-independent but E2F1-dependent apoptosis in these cancer cells. Because the death pathway induced by this combination does not depend on functional p53, this strategy for simultaneously targeting DNA and protein methylation may also be useful for the treatment of breast tumours harbouring p53 mutations.

In addition, tamoxifen has been used for the systemic treatment of patients with breast cancer for nearly three decades. Treatment success is primarily dependent on the presence of the ERα in the breast carcinoma. While about half of patients with advanced ERα-positive disease immediately fail to respond to tamoxifen, in the responding patients the disease ultimately progresses to a resistant phenotype. The possible causes for intrinsic and acquired resistance have been attributed to the pharmacology of tamoxifen, alterations in the structure and function of the ERα, the interactions with the tumour environment and genetic alterations in the tumour cells [40]. Therefore, understanding the role of ERα in the development and progression of hormone-unresponsive and receptor-dependent breast cancer is an important step in the development of future therapeutics. Recently, it has been suggested that ERα regulates E2F1 expression to mediate tamoxifen resistance [19]. Since E2F1 plays a dual role in cell survival/apoptosis, certainly, these findings are of relevance in the context of our study. Because TMCG/DIPY treatment positively influences E2F1-mediated cell death, we hypothesized that this combination might represent an attractive strategy to target overexpressed E2F1 in these tamoxifen resistant cells. The observation that TMCG/DIPY treatment was highly effective on MCF7TamR cells confirms this hypothesis and suggests that this combinational therapy could be extended to the treatment of patients with antiestrogen resistant breast cancers.

## Materials and Methods

### Reagents and Antibodies

TMCG was synthesised from catechin and by reaction with 3,4,5-trimethoxybenzoyl chloride [12]. DIPY, 4-hydroxytamoxifen, trichostatin, and *trans*-2-phenylcyclopropylamine (2PCPA) were obtained from Sigma-Aldrich (Madrid, Spain). Antibodies against the following proteins were used: β-Actin (Sigma; Monoclonal clone AC-15), E2F1 (Millipore, Madrid, Spain; Monoclonal clones KH20 and KH95), phospho-H2AX (Ser^139^) (Millipore; Monoclonal clone JBW301), acetyl-histone H4 (Ac-H4; Millipore, Polyclonal), HDAC1 (Millipore, Polyclonal), HDAC3 (Millipore, Monoclonal clone 3G6), MeCP2 (Millipore, Polyclonal), and RASSF1A (Abcam, Cambridge, UK; Monoclonal clone 3F3).

### Cell Cultures

MCF10 (a non-tumorigenic human breast epithelial cell line), the human breast cancer cell lines MCF7 and MDA-MB-231, and the murine breast cancer cell line 4T1 were obtained from the American Type Culture Collection. A tamoxifen-resistant MCF7 cell line (MCF7TamR) was developed from the parental line by maintaining cells in a medium containing 10 nM tamoxifen over 6 months. Resistant cells were characterized as described elsewhere [19]. Cells were maintained in the appropriate culture medium supplemented with 10% foetal calf serum and antibiotics. Cell viability was evaluated by a colorimetric assay for mitochondrial function using the 3-(4,5-dimethylthiazol-2-yl)-2,5-diphenyltetrazolium bromide (MTT; Sigma) cell proliferation assay. For this assay, cells were plated in a 96-well plate at a density of 1000–2000 cells/well. Compounds were added once at the beginning of each experiment.

### Apoptosis Assays

The induction of apoptosis was assessed by performing cytoplasmic histone-associated DNA fragmentation using a kit from Roche Diagnostics (Barcelona, Spain). Apoptosis was defined as the specific enrichment of mono- and oligonucleosomes in the cytoplasm and was calculated by dividing the absorbance of treated samples by the absorbance of untreated samples after correcting for the number of cells. The Hoechst staining method was also used to detect apoptosis. Replicate cultures of 1×10^5^ cells per well were plated in 6-well plates. The cells were subjected to the specified treatments for 72 h. After changing to fresh medium, the cells were incubated with 5 μL of Hoechst 33342 solution (Sigma) per well at 37°C for 10 min, then observed under a fluorescence microscope. Strong fluorescence was observed in the nuclei of apoptotic cells, while weak fluorescence was observed in non-apoptotic cells. Quantification of apoptotic cells was performed by counting the cells in four random fields in each well. When specified, analysis of apoptotic cells was performed using the terminal deoxynucleotidyl transferase-mediated dUTP nick-end labeling (TUNEL) staining kit following the manufacturer's instruction (Roche Diagnostics). Images of cells were taken using a fluorescence microscope.

### PCR Analysis

mRNA extraction, cDNA synthesis, and conventional and quantitative real-time PCR (qRT-PCR) were performed as previously described [10]. Primers were designed using Primer Express version 2.0 software (Applied Biosystems, Foster City, CA, USA) and synthesised by Invitrogen (Barcelona, Spain). The following primers for human genes were used: *β-Actin* (forward: 5′-AGA AAA TCT GGC ACC ACA CC-3′; reverse: 5′-GGG GTG TTG AAG GTC TCA AA-3′), *DNMT1* (forward: 5′-CCC CTG AGC CCT ACC GAA T-3′; reverse: 5′-CTC GCT GGA GTG GAC TTG TG-3′), and *RASSF1A* (forward: 5′-CAG ATT GCA AGT TCA CCT GCC ACT A-3′; reverse: 5′-GAT GAA GCC TGT GTA AGA ACC GTC CT-3′).

### DNA Methylation PCR Array

Genomic DNA was isolated using the Qiagen DNeasy tissue kit according to the manufacturer's instructions (Valencia, CA, USA). The differentially-methylated fractions of DNA were prepared using the Methyl-Profiler Enzyme kit, and DNA digests were analysed using the Human Breast Cancer Methyl-Profiler DNA Methylation PCR array kits according to the manufacturer's instructions (SABiosciences; Frederick, MD, USA). The complete list of genes is shown in Table S1.

### ChIP Assays

A chromatin immunoprecipitation (ChIP) assay was performed using the Magna ChIP^TM^ G kit from Millipore according to the manufacturer's instructions. Briefly, untreated and TMCG/DIPY-treated MDA-MB-231 cells were formaldehyde cross-linked, and the DNA was sheared by sonication to generate an average size of 300 to 3,000 bp. The chromatin was then incubated with anti-E2F1, anti-Ac-H4, anti-HDCA1, anti-HDAC3, anti-MeCP2 or mouse IgG antibodies. DNA from lysates prior to immunoprecipitation was used as a positive input control. After washing, elution, and DNA purification, the DNA solution (2 μl) was used as a template for qRT-PCR amplification using specific human primers. The following primer sequences were used for ChIP-PCR: *RASSF1A* region 1 (P1) (forward: 5′-GCT TCA GCA AAC CGG ACC-3′; reverse: 5′-CCG GAC GGC CAC AAC GA-3′), *RASSF1A* region 2 (P2) (forward: 5′-TGG GGT GTG AGG AGG GGA CGA-3′; reverse: 5′-AGA GCC GCG CAA TGG A-3′), *RASSF1A* region 3 (P3) (forward: 5′-GTT TCC ATT GCG CGG CTC T-3′; reverse: 5′-CTG GCT TTG GGC GCT AGC AAG-3′), and *GAPDH* (forward: 5′-CAA TTC CCC ATC TCA GTC GT-3′; reverse: 5′-TAG TAG CCG GGC CCT ACT TT-3′).

### Stealth RNA Transfection

Specific Stealth siRNAs for *RASSF1A* (HSS117376, HSS117377, and HSS174151) and E2F1 (HSS103015, HSS103016, and HSS103017 ) were obtained from Invitrogen and transfected into MDA-MB-231 cells using Lipofectamine 2000 (Invitrogen). Treatments were started 24 h after siRNA transfection. Stealth RNA negative control duplexes (Invitrogen) were used as control oligonucleotides, and the ability of the Stealth RNA oligonucleotides to knock down the expression of selected genes was analysed by confocal microscopy (RASSF1A) or western blot (E2F1) 24 h after shRNA transfection.

### Western blot Analysis

Whole cell lysates were collected by adding SDS sample buffer. After extensive sonication, samples were boiled for 10 min and subjected to SDS-PAGE. Proteins were then transferred to nitrocellulose membranes and analysed by immunoblotting (ECL Plus, GE Healthcare, Barcelona, Spain).

### Immunoprecipitation and MALDI-TOF Mass Spectroscopy

For immunoprecipitation assays, MDA-MB-231 cells (∼ 2.5×10^7^) were lysed in 500 μl of lysis buffer (50 mM Tris, pH 8.0, 300 mM NaCl, 0.4% NP40, and 10 mM MgCl_2_) supplemented with protease and phosphatase inhibitor cocktails (Sigma), 2.5 μM trichostatin (a potent deacetylase inhibitor), and 50 μM 2PCPA (an irreversible inhibitor of lysine-specific demethylase 1, LSD1). Cell extracts were cleared by centrifugation (20,000×g for 15 min) and then diluted with 500 μl of dilution buffer (50 mM Tris, pH 8.0, 0.4% NP40, and 2.5 mM CaCl_2_) supplemented with protease and phosphatase inhibitor cocktails, DNase I (Sigma), 2.5 μM trichostatin and 50 μM 2PCPA. The extracts were pre-cleared by 30-min incubations with 20 μl of PureProteome Protein G Magnetic Beads (Millipore) at 4°C with rotation. Then, the E2F1 antibody was covalently coupled to Dynabeads® (Invitrogen) and added to the pre-cleared extracts. After immunoprecipitation and elution, bound proteins were digested with trypsin according to standard procedures [41]. The data were recorded and processed with Agilent MassHunter Workstation Software to obtain the Peptide Mass Fingerprint (PMF). The PMF result mass spectra were searched against the E2F1 protein sequence with carbamidomethylation of cysteine as a fixed modification and methylation and acetylation of lysine residues, oxidation of methionine residues and phosphorylation of serine residues as variable modifications. The peptide mass tolerance was set to 50 ppm, and a maximum of three missed cleavages was considered.

### Microscopy

Confocal microscopy was carried out using a Leica TCS 4D confocal microscope (Wetzlar, Germany). For indirect immunofluorescence studies, preparation of the cells on glass slides were fixed with cold acetone for 5 min, and washed with PBS. The cells were incubated with 3% bovine serum albumin (BSA) for 20 min and then 2 h at room temperature with mouse-anti-human-RASSF1A [ab23950 specifically recognized the C1 domain (52–101 aa) of RASSF1A] or mouse-anti-human-phospho-H2AX (Ser139) [generated with KLH-conjugated, synthetic peptide (CKATQApSQEY) corresponding to aa 134–142 of human histone H2AX as immunogen] antibodies (both diluted 1∶200 in PBS containing 1% BSA). The cells were washed three times in PBS and incubated for 1 h at room temperature with Alexa Fluor Dyes (anti-mouse-633; Invitrogen) as secondary antibodies. After 3 washes with PBS, the cells were incubated with 0.01% 4′–6-diamidino-2-phenylidene (DAPI; Sigma) in water for 5 min. For antibody specificity, primary antibodies were replaced with specific IgGs (diluted 1∶200).

### Intracellular Adenosine

Control MDA-MB-231 cells or treated for 24 h with 10 µM DIPY (∼2.5×10^7^ cells each) were harvested, lysed in 500 ml 0.1 M HCl for 20 min, and centrifuged at 700×g for 10 min. Then, the supernatants were used for HPLC/MS/MS adenosine quantification assays. The peaks in the chromatographic runs of cell extracts were identified by comparing retention times, molecular ion peaks (m/z 268) and MS/MS characteristic fragments (m/z 136) with those of prepared pure adenosine standards. The concentrations of adenosine were calculated by comparing the peak areas with those of standards of known concentration. The analyses were carried out on an HPLC/MS system consisting of an Agilent 1100 Series HPLC (Agilent Technologies, Santa Clara, CA) connected to an Agilent Ion Trap XCT Plus mass spectrometer (Agilent Technologies) using an electrospray (ESI) interface. The mass spectrometer was operated in the positive mode. For each sample, 40 μl was injected onto a Zorbax Eclipse XDB-C18 HPLC column (5 μm, 150×4.6 mm, Agilent Technologies), thermostatted at 40°C. A binary gradient of mobile phase A (acetonitrile) and B (0.2% formic acid in water) was programmed at 0.50 mL/min. The proportions of mobile phase A:B was initially 10∶90 and switched to 100% mobile phase A from 0.01 to 1.8 min and switched back to 90% of mobile phase B from 3.5 min to 3.6 min and continued till 4.0 min.

### Statistical Analysis

In all experiments, the mean ± standard deviation (SD) values from three to five determinations in triplicate were calculated. Statistically significant differences were evaluated using Student's *t*-test. Differences were considered statistically significant at *P*<0.05.

## Supporting Information

Figure S1 The methionine cycle and its connections with other metabolic and cell survival pathways.
(PDF)

Figure S2 Bright field microscopy showing the differential effects of TMCG, DIPY, and TMCG/DIPY treatment on the growth and morphology of breast cancer cells.
(PDF)

Figure S3 TMCG/DIPY combination induces apoptosis in a tamoxifen resistant cell line (MCF7TamR). The images show the effect of TMCG/DIPY or 4-hydroxytamoxifen (4-OH-Tam) treatments on the apoptosis (upper panels) and morphology (lower panels) of MCF7 cells (parenteral and MCF7TamR) as determined by TUNEL and bright field microscopy, respectively. The histograms represent the apoptotic factor (assuming an apoptotic factor of 1 for MCF7 untreated cells) evaluated using a DNA fragmentation assay. Statistical values represent data from four replicate samples.
(PDF)

Figure S4 DIPY treatment increases the intracellular concentration of adenosine in MDA-MB-231 breast cancer cells.
(PDF)

Figure S5 Upregulation of RASSF1A in MDA-MB-231, after TMCG/DIPY treatment, is dependent of E2F1. E2F1 protein expression was monitored by western blot (WB). Semiquantitative reverse transcription-PCR was used to detect RASSF1A mRNA in siCN and siE2F1 MDA-MB-231 transfected cells treated for 3 days with 10 µM TMCG and 5 µM DIPY. β-actin was used as a loading control. Cell growth was monitored 4 days after transfection (3 days after TMCG/DIPY treatment) and apoptosis was determined by fluorescence microscopy after DNA staining with Hoechst 33342. In all cases, statistical values represent data from four replicate samples.
(PDF)

Table S1 qRT-PCR array analysis of the methylation status of 24 gene promoters that have been reported to be altered in a variety of breast cancers.
(PDF)
