# Supplementary figures and images for: Reactivation of the Tumour Suppressor RASSF1A in Breast Cancer by Simultaneous Targeting of DNA and E2F1 Methylation
Source: PLoS One. 2012 Dec 14;7(12):e52231. doi: 10.1371/journal.pone.0052231 (PMC3522638; doi:10.1371/journal.pone.0052231)

Figure S1. Montenegro et al.

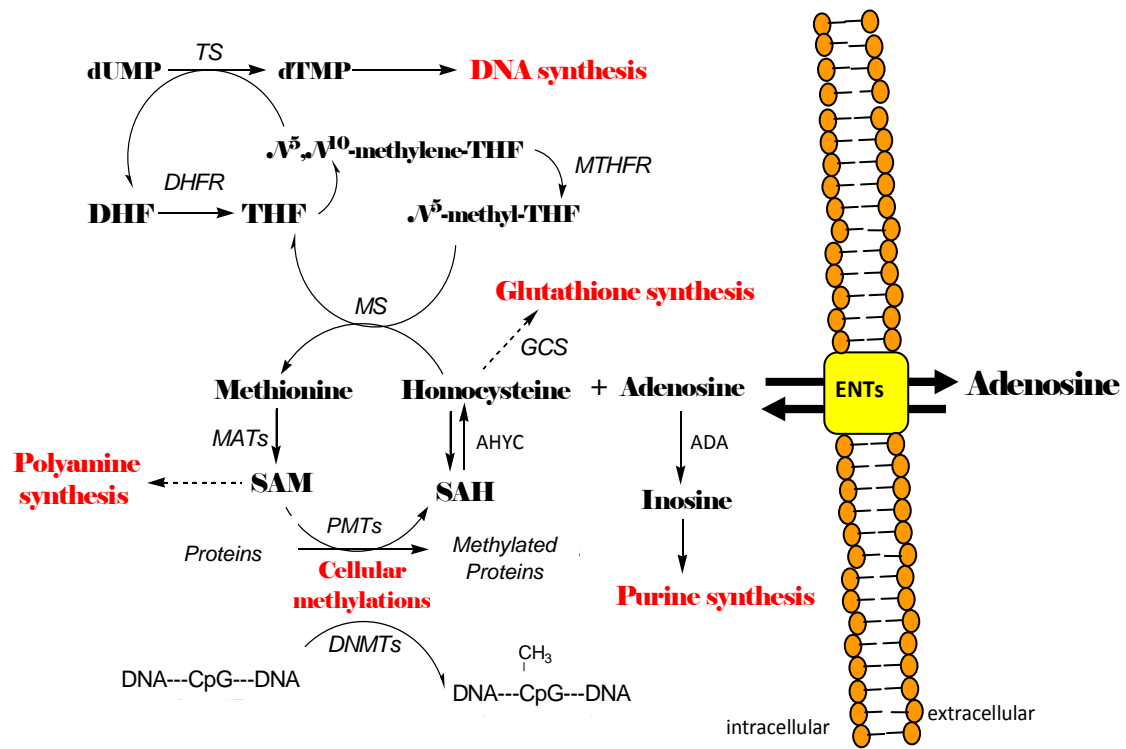

Supplement: Figure S1 — The methionine cycle and its connections with other metabolic and cell survival pathways. (PDF) [file pone.0052231.s001.pdf]

**Figure S2. Montenegro et al.**

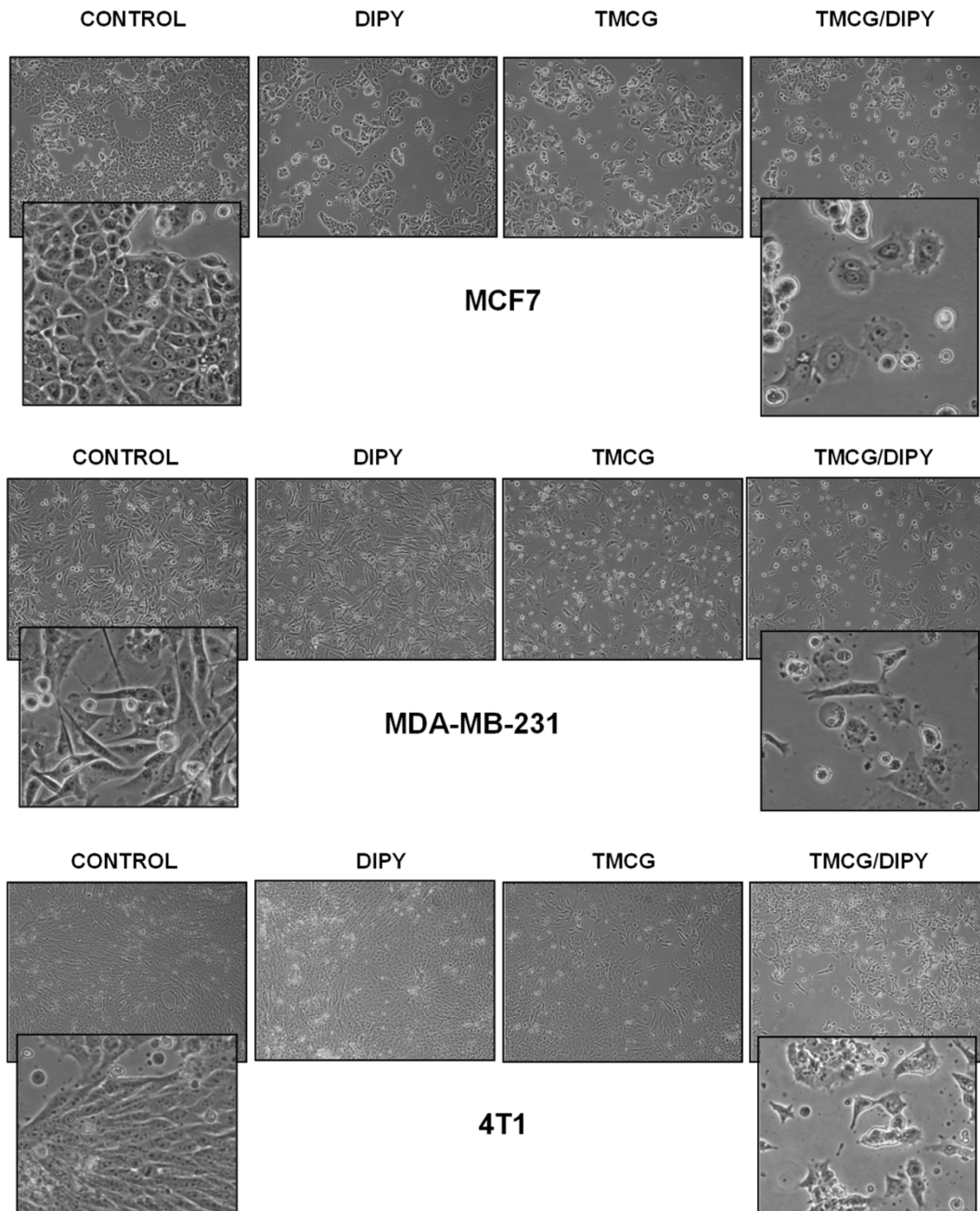

Supplement: Figure S2 — Bright field microscopy showing the differential effects of TMCG, DIPY, and TMCG/DIPY treatment on the growth and morphology of breast cancer cells. (PDF) [file pone.0052231.s002.pdf]

Figure S3. Montenegro et al.

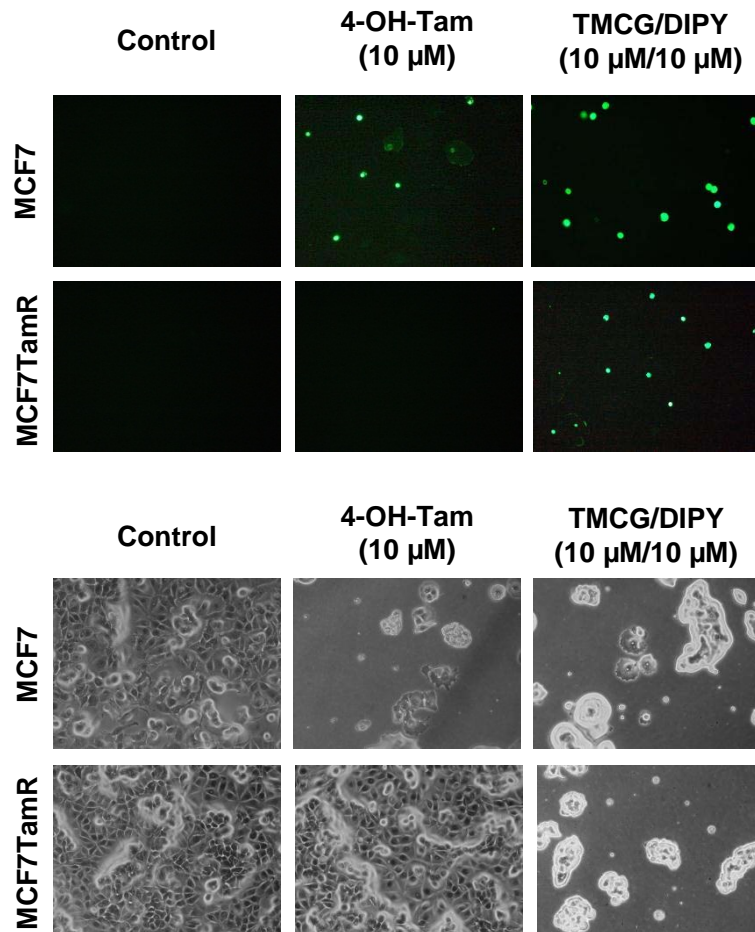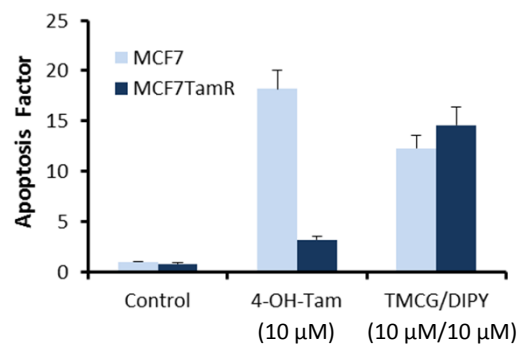

Supplement: Figure S3 — TMCG/DIPY combination induces apoptosis in a tamoxifen resistant cell line (MCF7TamR). The images show the effect of TMCG/DIPY or 4-hydroxytamoxifen (4-OH-Tam) treatments on the apoptosis (upper panels) and morphology (lower panels) of MCF7 cells (parenteral and MCF7TamR) as determined by TUNEL and bright field microscopy, respectively. The histograms represent the apoptotic factor (assuming an apoptotic factor of 1 for MCF7 untreated cells) evaluated using a DNA fragmentation assay. Statistical values represent data from four replicate samples. (PDF) [file pone.0052231.s003.pdf]

**Figure S4. Montenegro et al.**

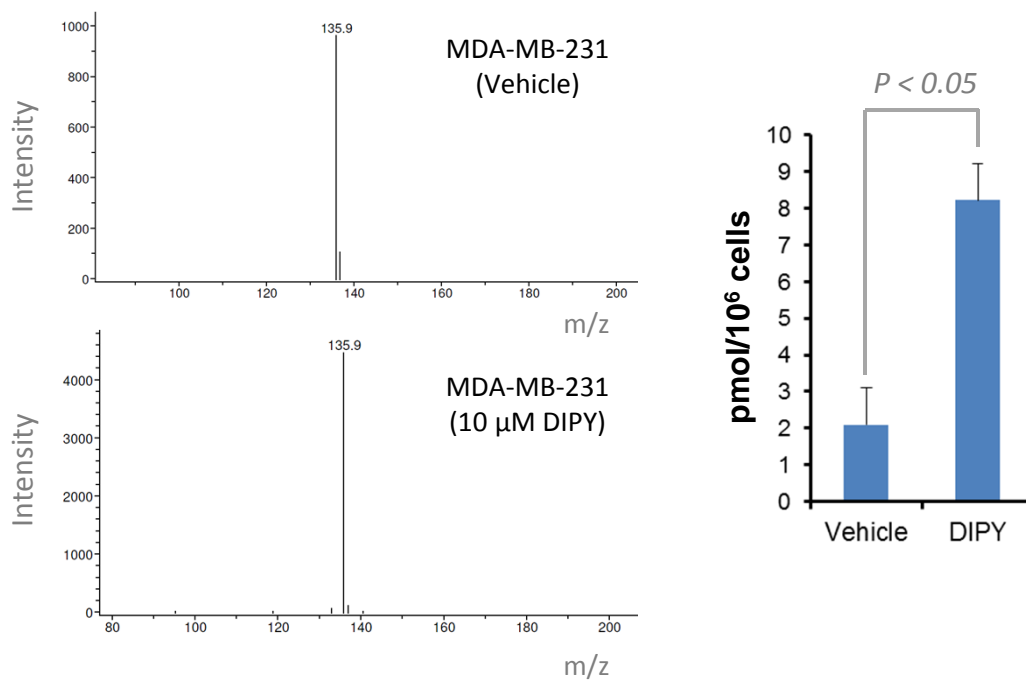

Supplement: Figure S4 — DIPY treatment increases the intracellular concentration of adenosine in MDA-MB-231 breast cancer cells. (PDF) [file pone.0052231.s004.pdf]

Figure S5. Montenegro et al.

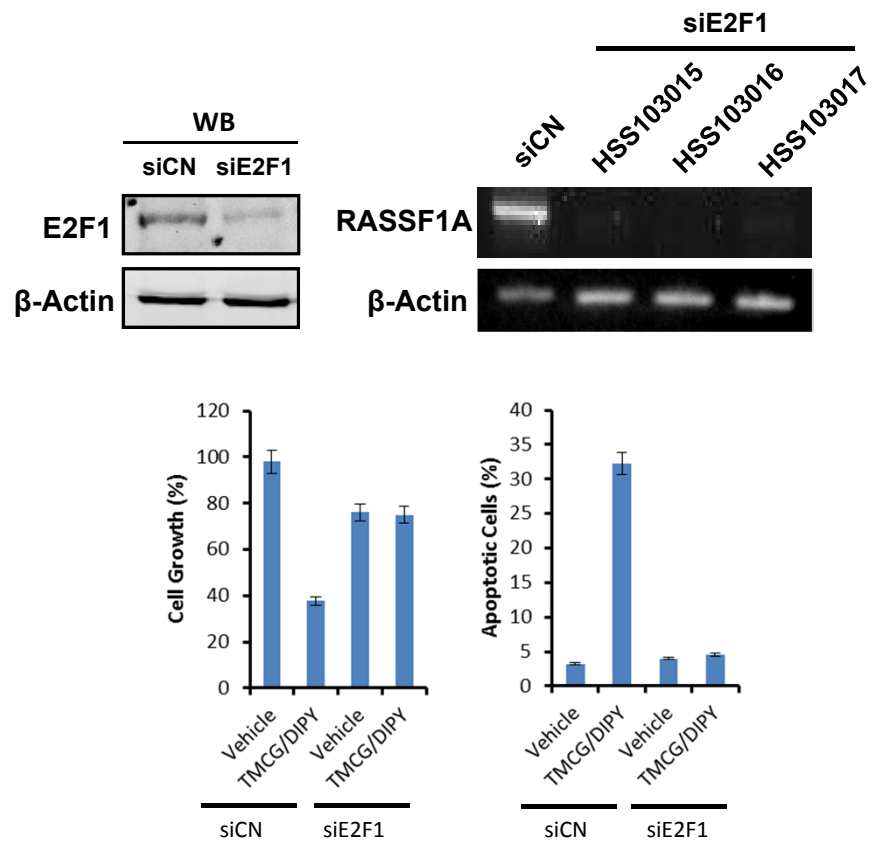

Supplement: Figure S5 — Upregulation of RASSF1A in MDA-MB-231, after TMCG/DIPY treatment, is dependent of E2F1. E2F1 protein expression was monitored by western blot (WB). Semiquantitative reverse transcription-PCR was used to detect RASSF1A mRNA in siCN and siE2F1 MDA-MB-231 transfected cells treated for 3 days with 10 µM TMCG and 5 µM DIPY. β-actin was used as a loading control. Cell growth was monitored 4 days after transfection (3 days after TMCG/DIPY treatment) and apoptosis was determined by fluorescence microscopy after DNA staining with Hoechst 33342. In all cases, statistical values represent data from four replicate samples. (PDF) [file pone.0052231.s005.pdf]
