# Supplementary material for: Reactivation of the Tumour Suppressor RASSF1A in Breast Cancer by Simultaneous Targeting of DNA and E2F1 Methylation
Source: PLoS One. 2012 Dec 14;7(12):e52231. doi: 10.1371/journal.pone.0052231 (PMC3522638; doi:10.1371/journal.pone.0052231)
Supplement: Table S1 — qRT-PCR array analysis of the methylation status of 24 gene promoters that have been reported to be altered in a variety of breast cancers. (PDF) [file pone.0052231.s006.pdf]

Table S1. Complete list of array methylation results

Gene Symbol	MCF10A			MDA-MB-231						MCF7					
	Untreated			Untreated			TMCG/DIPY			Untreated			TMCG/DIPY		
	HM	UM	IM	HM	UM	IM	HM	UM	IM	HM	UM	IM	HM	UM	IM
ADAM23	n/a	n/a	n/a	n/a	n/a	n/a	n/a	n/a	n/a	n/a	n/a	n/a	23.36%	22.65%	54.00%
CDKN2A	n/a	n/a	n/a	76.08%	23.92%	0.00%	51.18%	42.92%	5.90%	0.02%	99.98%	0.00%	31.51%	0.65%	67.84%
BRCA1	3.04%	96.96%	0.00%	0.02%	0.02%	99.95%	0.66%	14.52%	84.81%	0.01%	0.01%	99.98%	0.08%	19.11%	80.81%
CCNA1	0.00%	100.00%	0.00%	25.37%	74.63%	0.00%	0.00%	0.02%	99.98%	17.79%	82.21%	0.00%	0.08%	99.92%	0.00%
CCND2	n/a	n/a	n/a	n/a	n/a	n/a	0.00%	100.00%	0.00%	n/a	n/a	n/a	n/a	n/a	n/a
CDH1	88.63%	11.37%	0.00%	4.67%	95.33%	0.00%	0.04%	99.96%	0.00%	0.12%	99.88%	0.00%	0.05%	99.95%	0.00%
CDH13	47.91%	37.80%	14.29%	87.79%	12.21%	0.00%	82.57%	17.43%	0.00%	n/a	n/a	n/a	91.31%	8.69%	0.00%
CDKN1C	0.14%	99.86%	0.00%	1.31%	98.69%	0.00%	10.55%	8.77%	80.68%	3.26%	45.33%	51.41%	0.15%	99.85%	0.00%
CDKN2A	0.74%	99.26%	0.00%	n/a	n/a	n/a	52.06%	22.48%	25.46%	n/a	n/a	n/a	0.07%	99.93%	0.00%
ESR1	n/a	n/a	n/a	14.63%	85.37%	0.00%	14.35%	14.21%	71.45%	1.12%	98.88%	0.00%	0.12%	99.88%	0.00%
GSTP1	n/a	n/a	n/a	0.00%	100.00%	0.00%	0.30%	99.70%	0.00%	24.46%	1.34%	74.20%	0.12%	0.02%	99.86%
HIC1	1.54%	98.46%	0.00%	94.36%	5.64%	0.00%	0.03%	99.97%	0.00%	95.76%	4.24%	0.00%	0.01%	99.99%	0.00%
MGMT	80.25%	19.75%	0.00%	0.49%	99.51%	0.00%	0.08%	99.92%	0.00%	0.00%	100.00%	0.00%	43.32%	1.10%	55.59%
PRDM2	93.91%	6.09%	0.00%	0.05%	99.95%	0.00%	40.21%	11.01%	48.79%	0.09%	99.91%	0.00%	34.69%	8.73%	56.59%
PTEN	62.01%	37.99%	0.00%	0.01%	99.99%	0.00%	0.03%	99.97%	0.00%	0.00%	100.00%	0.00%	0.00%	17.59%	82.41%
PTGS2	0.12%	99.88%	0.00%	0.09%	99.91%	0.00%	0.10%	99.90%	0.00%	0.00%	100.00%	0.00%	0.06%	99.94%	0.00%
PYCARD	0.30%	99.70%	0.00%	0.02%	99.98%	0.00%	0.68%	38.10%	61.22%	0.03%	99.97%	0.00%	31.47%	15.17%	53.37%
RASSF1	0.03%	99.97%	0.00%	87.98%	12.02%	0.00%	26.88%	1.07%	72.06%	90.37%	9.63%	0.00%	29.59%	9.39%	61.02%
SFN	87.76%	12.24%	0.00%	0.59%	99.41%	0.00%	91.23%	8.77%	0.00%	0.38%	99.62%	0.00%	87.68%	12.32%	0.00%
SLIT2	0.00%	100.00%	0.00%	0.12%	99.88%	0.00%	0.00%	100.00%	0.00%	n/a	n/a	n/a	0.00%	100.00%	0.00%
THBS1	0.09%	99.91%	0.00%	0.02%	99.98%	0.00%	1.81%	19.37%	78.82%	0.02%	99.98%	0.00%	0.11%	99.89%	0.00%
TNFRSF10C	89.73%	10.27%	0.00%	0.81%	99.19%	0.00%	31.96%	1.93%	66.11%	0.32%	99.68%	0.00%	1.94%	9.80%	88.26%
TNFRSF10D	13.31%	13.31%	73.38%	0.39%	99.61%	0.00%	0.19%	99.81%	0.00%	n/a	n/a	n/a	0.04%	99.96%	0.00%
TP73	23.24%	76.76%	0.00%	98.53%	1.47%	0.00%	0.07%	28.70%	71.23%	98.87%	1.13%	0.00%	4.02%	14.90%	81.08%

HM = hypermethylated; UM = unmethylated; IM = intermediately methylation; n/a = below detectable limits.
